# Successful treatment of aortic dissection during sorafenib therapy for hepatocellular carcinoma

**DOI:** 10.1002/ccr3.1674

**Published:** 2018-07-01

**Authors:** Atsunori Tsuchiya, Masahiro Ogawa, Yusuke Watanabe, Naruhiro Kimura, Kazunao Hayashi, Takeshi Suda, Shuji Terai

**Affiliations:** ^1^ Division of Gastroenterology and Hepatology Graduate School of Medical and Dental Science Niigata University Chuo‐ku, Niigata Japan; ^2^ Department of Gastroenterology and Hepatology Uonuma Institute of Community Medicine Niigata Medical and Dental Hospital Niigata Japan

**Keywords:** sorafenib, aortic dissection, vascular endothelial growth factor pathway inhibitor

## Abstract

Our case highlights the need for caution during vascular endothelial growth factor pathway inhibitor (VPI) therapy and for the occurrence of aortic dissection. If Stanford classification type A aortic dissection occurs during VPI therapy, surgical intervention should be considered to prevent cardiac tamponade if the patient's clinical condition permits it.

Sorafenib, a multikinase inhibitor often used to treat advanced hepatocellular carcinoma (HCC), can cause adverse effects, such as hand–foot syndrome and hypertension.^1^ We report a case of successfully treated aortic dissection during sorafenib therapy. A 65‐year‐old man (chronic hepatitis due to hepatitis B virus) had a large HCC with portal vein invasion and liver metastasis (Barcelona clinic liver cancer stage C). Eleven months after HCC treatment by hepatic arterial infusion chemotherapy using diamminedichloroplatinum (DDP‐H), daily administration of 400 mg sorafenib was commenced. Two weeks after sorafenib therapy, an angiotensin II receptor blocker was started as his blood pressure increased to 152/82 (128/54 on admission). Two months later, the patient developed widespread aortic dissection (Stanford classification type A) (Figure 1; white arrows). Sorafenib was stopped after this event. To prevent cardiac tamponade, ascending aorta replacement was performed, and the patient survived for 34 months after the operation by systemic chemotherapy using low‐dose cisplatin and 5‐fluorouracil. Using a Japanese Adverse Drug Event Report database, Oshima et al reported that 49 of 16 441 patients (0.3%) treated with systemic vascular endothelial growth factor pathway inhibitors (VPIs) developed aortic dissection (30 times higher than in subjects not exposed to VPIs). This includes 11 of 4975 (0.22%) patients treated with sorafenib.^2^ While aortic dissection is rare, it is likely caused by an increase in blood pressure and delayed healing of damaged endothelium. Patients on VPI therapy should be monitored closely for elevation in blood pressure and aortic dissection.

**Figure 1:**
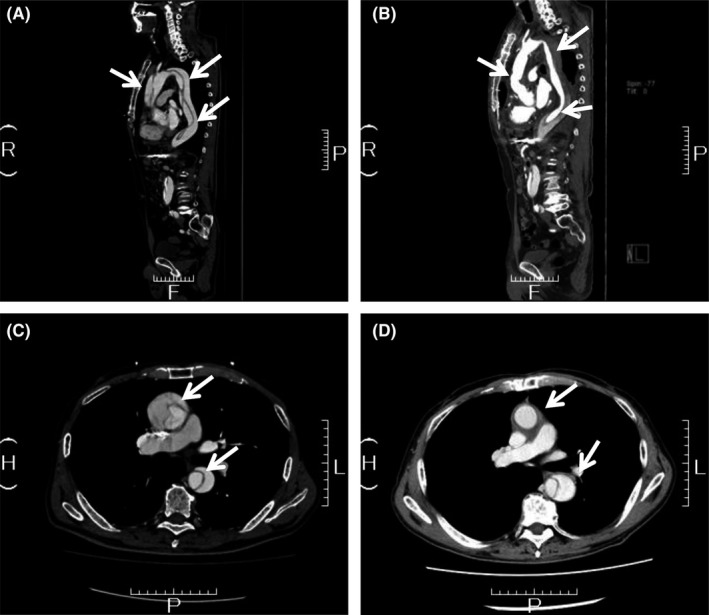
Sagittal (A and B) and transverse (C and D) views of the abdominal CT demonstrating widespread aortic dissection from the chest lesion to the abdominal lesion (A and C; before operation, B and D; 2 mo after operation)

## CONFLICT OF INTEREST

The authors have declared that no conflict of interest exists.

## INFORMED CONSENT

Obtained for the publication of this case study.

## AUTHORSHIP

All authors are doctors in charge, and all authors contributed to the writing of this manuscript.
